# HydroNanoConstruct: A Web Application for Digital Construction, Crystal Growth Investigation, and Atomistic Descriptor Calculation of Hydrated Metal Oxide Nanoparticles Powered by the EosCloud Platform

**DOI:** 10.1021/acs.jcim.5c01889

**Published:** 2025-12-29

**Authors:** Panagiotis D. Kolokathis, Anastasios Sourpis, Dimitris Mintis, Andreas Tsoumanis, Georgia Melagraki, Milica Velimirovic, Iseult Lynch, Antreas Afantitis

**Affiliations:** † NovaMechanics MIKE, Piraeus 18545, Greece; ‡ 655868 Entelos Institute, Larnaca 6059, Cyprus; § 443801 NovaMechanics Ltd, Nicosia 1070, Cyprus; ∥ Division of Physical Sciences and Applications, Hellenic Military Academy, Vari 16672, Greece; ⊥ Flemish Institute for Technological Research (VITO), Mol 2400, Belgium; # School of Geography, Earth and Environmental Sciences, 1724 University of Birmingham, Birmingham B15 2TT, U.K.; ¶ Department of Pharmacy, Frederick University, Nicosia 1036, Cyprus

## Abstract

HydroNanoConstruct is a web application with a graphical user interface (GUI) that enables the digital construction and analysis of hydrated metal/metalloid oxide (ΜΟ) nanoparticles (NPs). The tool supports (a) building hydrated ΜΟ NPs from crystallographic information files (CIFs) of MOs by adding hydrogen cations, hydroxyl anions, and water molecules to the surface atoms, preserving the bulk material’s coordination numbers for the metal/metalloid atoms, (b) performing energy minimization on the digitally constructed hydrated MO NPs to obtain realistic structures, (c) embedding the hydrated MO NP into a simulation box filled with water molecules to incorporate solvent effects, and (d) running multiple molecular dynamics and energy minimization cycles to compute atomistic descriptors, including surface energy (in vacuum and in water) and the potential energy of the bulk material. The tool is freely available via the EosCloud Platform at https://eoscloud.entelos.eu/ssbd4chem/nanoconstruct/.

## Graphical abstract

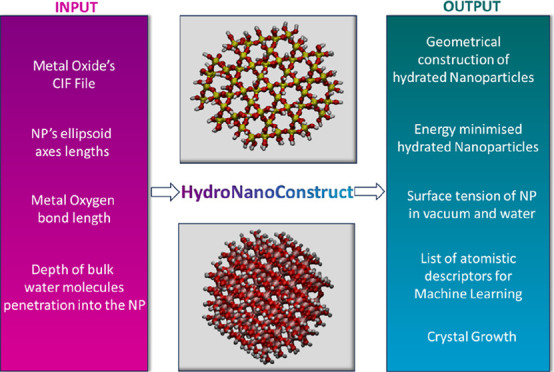

## 1 Introduction

Nanoparticles (NPs) have attracted significant attention due to their unique properties which often differ from those of their corresponding bulk materials.^1–4^ Ιn silico tools^5,6^ are frequently used to calculate the properties of various NPs and to screen the most promising ones, prior to their synthesis and experimental validation. However, the digital construction of NPs is still one of the most challenging steps in the in silico process despite the availability of several supporting tools. The CHARMM GUI Nanomaterial modeler^7^ is one such tool that can be used to construct NPs but it currently supports only a limited number of materials (e.g., Al_2_O_3_, Fe_2_O_3_, Cr_2_O_3_, CaO, MgO and NiO among the class of metal oxides). Another tool, called NanoCrystal,^8^ offers a more generic approach allowing users to upload crystallographic information files (CIFs) to build spherical stoichiometric and polyhedral based on Miller indices NPs using a geometric approach. This tool also requires user-provided macroscopic surface energies for each Miller index in order to predict the particle’s shape and describe its crystal growth via the Wulff construction method^9,10^ which is most accurate and meaningful for large particles, where macroscopic surface energies can be reliably applied. Another tool, called NanoConstruct^11,12^ which is also generic and available through the Enalos Cloud Platform,^13,14^ digitally constructs ellipsoid NPs in vacuum and applies energy minimization to generate realistic NP structures. This is important because surface atoms typically exhibit reduced coordination numbers compared to bulk atoms, making purely geometrical constructions less accurate. All of the above-mentioned tools are limited to modeling NPs in vacuum (or in air which can be approximated as vacuum due to its low density) or NPs that do not react with water. However, many NPs, especially metal/metalloid oxides (MOs), undergo structural and chemical changes when embedded in liquid water. In such cases, the coordination number of surface metal atoms is often preserved to match that of the bulk phase through interactions with water molecules, hydroxyl groups, or hydrogen ions. In contrast, a MO NP in vacuum or air generally retains its stoichiometry (provided it is neutrally charged), while a hydrated MO NP maintains the coordination number of its metal atoms consistent with that of the bulk material.^4,15–26^ HydroNanoConstruct aims to simplify the digital construction of MO NPs in water and to generate realistic NP structures through a user-friendly web application.

## 2 Key Functionalities

HydroNanoConstruct allows users to upload CIF Files of MOs, which can be accessed through the Crystallography Open Database (COD).^27^ This generic tool can support Safe and Sustainable by Design (SSbD) strategies for the next generation of nanomaterials.^28^ For example, HydroNanoConstruct can be used to investigate the nucleation process of MO NPs by calculating the free energy of the NP–water system (see eq 1), thereby enabling the identification of potential nucleation barriers. Unlike classical nucleation theory,^29^ which lacks atomistic resolution, relying instead on bulk surface tension values and an estimated number of atoms based on bulk density, HydroNanoConstruct enables accurate calculation of surface tension and NP density through atomistic simulations,^30^ explicitly accounting for atomistic effects. In particular, surface energies of spherical Au and Pt NPs in vacuum have already been computed,^30^ showing a strong size dependence. HydroNanoConstruct facilitates the extension of such calculations to MOs in water. Generally, the surface energy is considered to be a positive number for bulk materials. A negative surface energy would suggest that atoms at the surface are thermodynamically more stable than those in the interior, indicating bulk instability.^31,32^ Such a scenario could imply that particles might be more stable in the nanoscale form than in their bulk phase.^31^ HydroNanoConstruct aims to shed light on these surface tension differences between bulk materials and their corresponding NPs thereby helping to explain why NPs are often more stable than the bulk phase.

In the following sections, we describe an algorithm for the digital construction of realistic MO NPs in aqueous environments. This algorithm can be then applied to calculate the surface tension of the MO NPs, assess their stability in water, and investigate their crystal growth pathways (e.g., either directly through the web application or externally using the generated output files). Additionally, a set of atomistic descriptors is generated (see Supporting Information), which can be used to enrich experimental data sets and support the development of machine learning models. HydroNanoConstruct is structured into four distinct stages, and its graphical user interface (GUI) is shown in Figure 1. The GUI integrates JSmol, a JavaScript-based molecular viewer derived from Jmol,^33^ to enable interactive visualization and illustration of the created structures.

**Figure 1:**
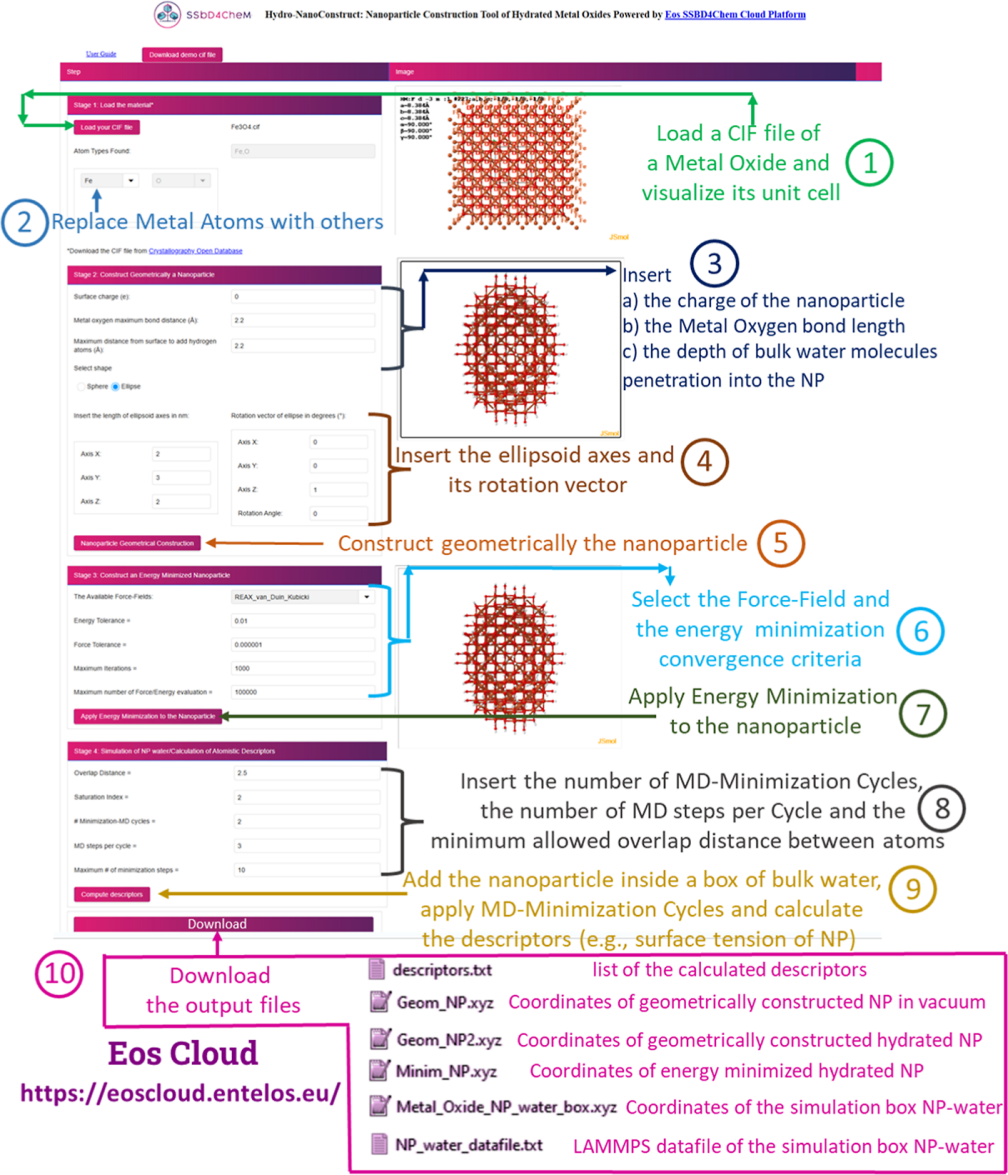
Graphical User Interface of the HydroNanoConstruct Web Application showing the required input files, the four main stages of digital NP construction, and the generated output files.

## 3 Methodology

### 3.1 Algorithm for the Geometrical Construction of Hydrated Metal Oxide Nanoparticles

As mentioned in literature,^15–25^ metal oxides (MOs) react with water forming surface hydroxyl groups or surface chemically bonded water molecules (≡M–OH, ≡M–OH_2_, ≡M–OH-M≡). These surface groups give a hydrophilic character to the MOs and allow the physical adsorption of other water molecules. To construct a hydrated MO NP, one must first generate a stoichiometrical NP (i.e., in vacuum or air) by following the procedure already described in the literature.^11,12^ This procedure (see Figures S1 and S2 of the Supporting Information) involves the creation of a simulation box of the bulk material and the removal of the atoms that are located beyond the chosen NP’s radius. During this procedure, the neighboring (i.e., within metal–oxygen bond distance) oxygen atoms of each metal atom are stored. Next, the oxygen atoms that were not initially included in the created NP but were bonded to metal atoms belonging to the NP in the original bulk phase (i.e., before the removal of the atoms that were beyond the NP’s radius) are added back to NP. This addition of oxygen atoms preserves the coordination number of the metal atoms (i.e., the number of atoms bonded to each metal atom), keeping it equal to that in the bulk phase. For each oxygen atom that is added and that was not part of the initial NP, a charge of −2e is added to the overall charge of the NP. However, this procedure may lead to oxygen atoms that are also undercoordinated. To make the NP structure more realistic, a termination of these undercoordinated oxygen atoms is necessary and so, hydrogen atoms are added too. The number of the hydrogen atoms that will be added (i.e., + 1e is added to the NP’s charge for every hydrogen atom added), determines the overall charge of the NP which is also known to depend on the pH of its aquatic environment.^34,35^

A special algorithm has been developed to place the hydrogen atoms (i.e., find the positions of the added hydrogen atoms as presented in Figure S2). The algorithm classifies metal atoms into two groups: those with four or fewer coordination number/bonds, and those with higher coordination number. In addition, hydrogen atoms are classified as (a) hydroxyl hydrogen atoms, (b) bridging oxygen’s hydrogen atoms and (c) water hydrogen atoms. This classification of atoms is illustrated in Figure 2. If an oxygen atom is bonded to a 4 (or less) -coordinated metal atom, and is not bonded to another metal atom (i.e., an unpaired oxygen atom), it is considered undercoordinated, and a hydrogen atom is added to chemically terminate it. If an oxygen atom is bonded to a 5 (or more) -coordinated metal atom, and is not bonded to another metal atom, then one hydrogen atom is added to chemically terminate it until the four oxygen atoms bonded to the metal atom are paired or terminated with a hydrogen atom. For the remaining oxygen atoms, two hydrogen atoms are added to them (i.e., water hydrogen atoms).

**Figure 2:**
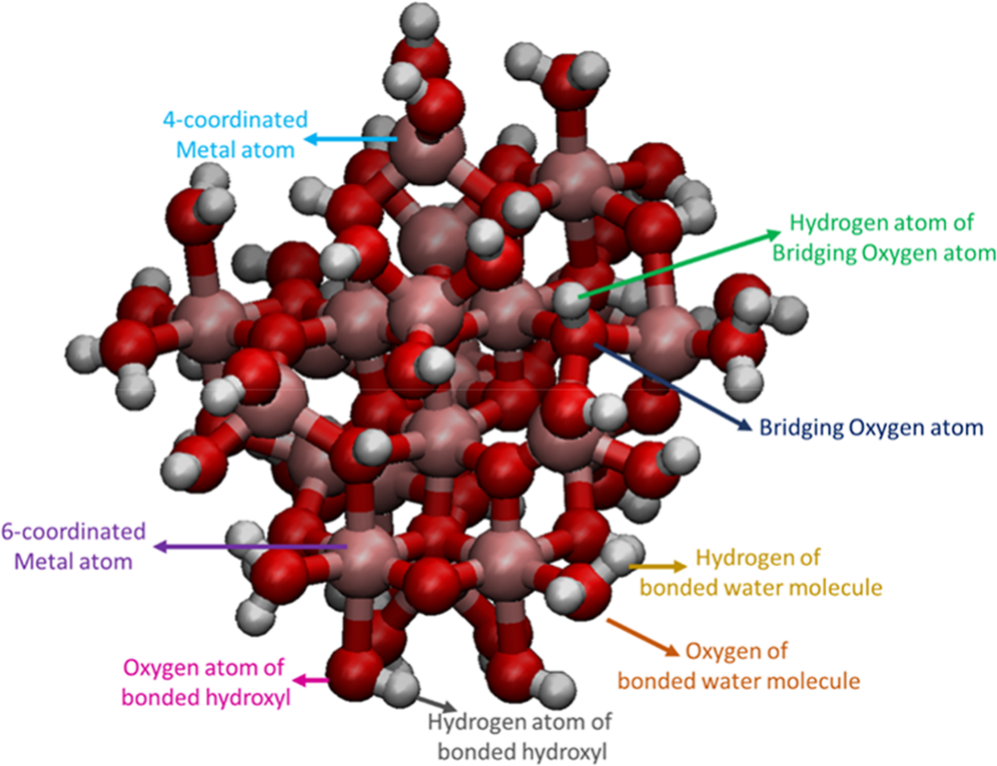
Energy-minimized Fe_3_O_4_ (magnetite) NP with a diameter of 1 nm. Iron, oxygen, and hydrogen atoms are represented by pink, red, and gray colors, respectively. Arrows indicate the distinct types of metal, oxygen, and hydrogen atoms present in the structure. Molecular visualization of Fe_3_O_4_ (magnetite) NP was performed using VMD (visual molecular dynamics) developed by the Theoretical and Computational Biophysics Group in the Beckman Institute for Advanced Science and Technology at the University of Illinois at Urbana–Champaign.^36^

In addition to the hydroxyl and water hydrogen atoms, there are also the bridging oxygen’s hydrogen atoms. Bridging oxygen atoms are those bonded to two metal atoms. The bridging oxygen’s hydrogen atoms are considered acidic (i.e., Bronsted acid site)^37^ and are also added after the addition of the hydroxyl and water hydrogen atoms. They are placed within a distance from the surface where the surface is considered to be accessible to the solvent (e.g., for porous materials this distance can be equal to the radius of the NP). All the hydrogen atoms are added with a direction perpendicular to the NP’s surface to avoid any overlap with other atoms of the NP. Next, to construct a NP with a user-specified electric charge, the previously added hydrogen atoms are removed according to the following order. First, the bridging oxygen’s hydrogen atoms are removed from the inner to the outer ones. If no more bridging oxygen’s hydrogen atoms remain to be removed, one hydrogen atom from each pair of the water hydrogen atoms (i.e., a hydroxyl remains) is removed. Finally, if only hydroxyl hydrogen atoms remain, hydrogen atoms are removed from them until the NP reaches the user defined charge. Based on the algorithm described above, the user must provide the maximum allowed metal oxygen bond length and the depth of bulk water molecules and hydroxyls penetration into the NPs.

The methodology described above produces structures that are consistent with those found in previous computational studies^38^ (e.g., the predicted structures of iron monomers and dimers) and it also agrees with density functional theory (DFT) calculations available in the literature.^21–26,39,40^ Specifically, Ivanov and Lyubartsev^24^ considered high reactivity to 4-coordinated Ti atoms and this approach has been incorporated into the present algorithm for all of the tetrahedral (i.e., 4-coordinated) metal/metalloid atoms. This is also in agreement with the 4-coordinated Silicon atoms and the water silanol interactions on the amorphous silica surface as investigated using DFT.^25^ Furthermore, Agosta et al.^39^ used DFT to study the interactions of water with TiO_2_ surfaces and found that water molecules are bonded to 5 and 6 coordinated Titanium atoms while hydroxyl groups are formed on the 4 coordinated Titanium atoms. They also investigated the addition of hydrogen atoms to bridging oxygen atoms

### 3.2 Construction of Energy Minimized Hydrated Metal Oxide Nanoparticles

The algorithm described above (see Figure S2 for more details) is limited to the geometrical construction of a hydrated MO NP and is related to stages 1 and 2 as illustrated in the GUI of HydroNanoConstruct (see Figure 1). To ensure that HydroNanoConstruct produces realistic NPs, two additional stages, stages 3 and 4, are applied (see Figure 1). In stage 3, energy minimization is performed on a hydrated MO NP in vacuum (i.e., without surrounding water solvent molecules). The absence of solvent significantly reduces the computational cost. Stage 3 corrects any less stable configuration^39^ by allowing the rearrangement of all atoms. In stage 4, water molecules are added to the simulation box to represent the solvent, which increases the computational cost. To prevent overlaps between the added water molecules and the NP atoms, a user defined variable is introduced. This variable defines the minimum allowed distance between water and NP atoms.

Despite the computational cost, stage 4 (see Figure 3) can provide even more realistic NP structures than stage 3 because it also includes the interaction between the NP and the surrounding water molecules (e.g., the hydroxylation of metal oxide surfaces^16^). This interaction may result in the removal or addition of hydrogen atoms, hydroxyl groups and water molecules from or onto the NP (e.g., the presence of bulk water molecules may accelerate the displacement of hydrogen atoms of the NP to the neighboring hydroxyl/water groups, leading to the formation of hydronium). The dimensions of the simulation box are selected so that (a) the NP is fully contained within the box and (b) NP atoms do not interact with the periodic images of the NP atoms (assuming a cutoff distance 14 Å). This simulation box is constructed by replicating a smaller equilibrated liquid water box with an edge length of 18.62 Å ensuring the above criteria are met. Overlaps with the NP water molecules are removed and an additional box is added in each direction to further ensure that the NP atoms do not interact with the NP’s atoms periodic images. Any overlapping water that has at least one atom closer than a specified distance (i.e., the minimum allowed overlap distance) to the energy minimized NP atoms is removed. In all calculations of HydroNanoConstruct, a cutoff distance is applied to prevent interactions between the NP’s atoms and the periodic images of the all NP atoms. For example, when the REAXFF force field is selected, the Wolf method^41^ is used for the calculation of the electrostatic interactions.

**Figure 3:**
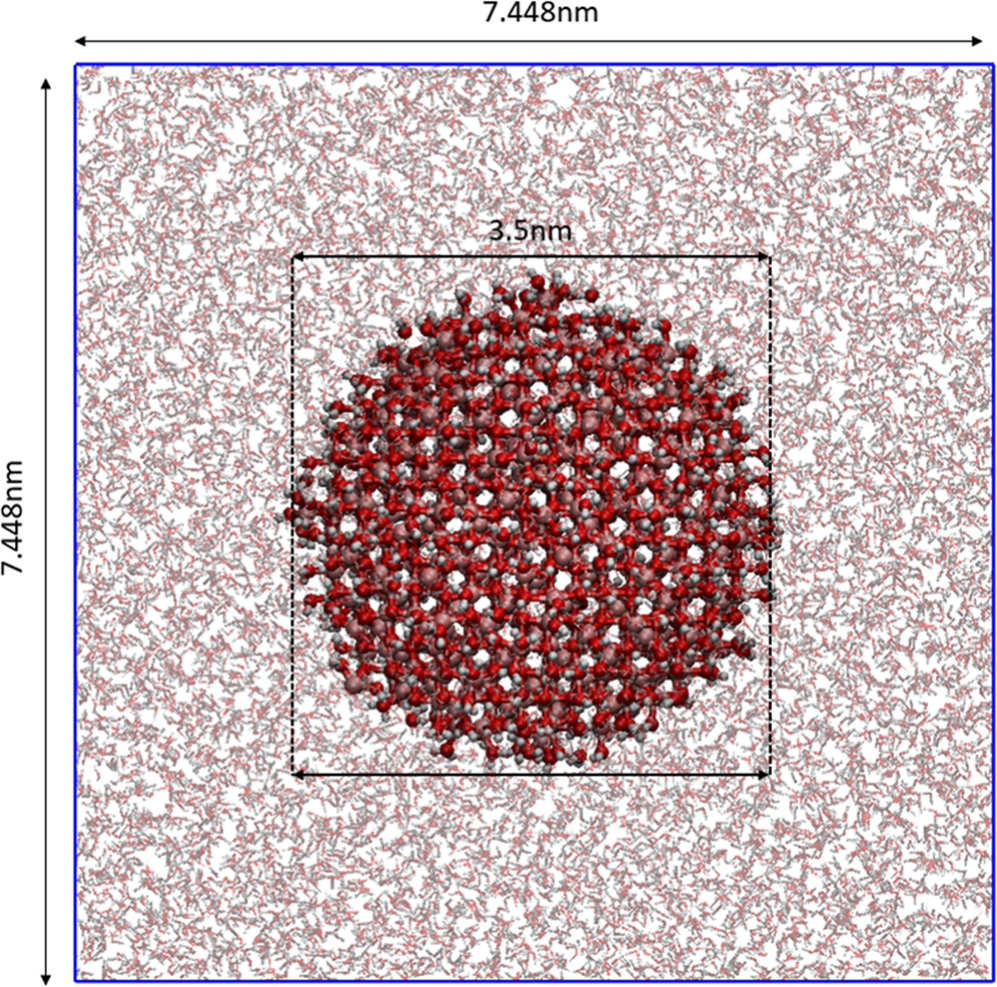
An Fe_3_O_4_ NP surrounded by bulk water after 400 MD-minimization cycles. The water molecules in the water bulk phase are illustrated with pale lines while the atoms of the Fe_3_O_4_ NP and its bonded water, hydroxyl groups, hydrogen atoms are shown as intense spheres and bonds. Iron, oxygen and hydrogen atoms are illustrated with pink, red and gray colors, respectively. Molecular visualization of Fe_3_O_4_ (magnetite) NP was performed using VMD (visual molecular dynamics) developed by the Theoretical and Computational Biophysics Group in the Beckman Institute for Advanced Science and Technology at the University of Illinois at Urbana–Champaign.^36^

To help the system overcome any probable local minima in which it may be trapped because of the initial configuration generated by the algorithm, HydroNanoConstruct enables the application of molecular dynamics (MD) and energy minimisation cycles (see Figure S3). Stages 3 and 4 use atomistic force fields (FFs) such as the REAXFF^42,43^ and COMB^44^ and other FFs available from the OPENKIM database.^45^ To allow further simulations, HydroNanoConstruct enables users to download the generated configurations files in LAMMPS^46–48^ format (e.g., LAMMPS datafiles) and continue the simulations externally (for more details see the procedure illustrated in Figure 1). To further validate the algorithm for the geometrical construction of NPs, the energy minimization calculations using reactive force fields^42^ were performed for SiO_2_ (a-quartz) and Fe_3_O_4_ (magnetite) NPs^49,50^ where excellent agreement was found (see Figures S4 and S5). A more detailed description for the validation of HydroNanoConstruct’s algorithm’s is provided in the Supporting Information.

## 4 Conclusions

This work presents HydroNanoConstruct, a web application for the digital construction of energy minimized hydrated Metal Oxide and Metalloid Oxide NPs using crystallography information file (CIF), such as those of the Crystallography Open Database (COD) as input. The algorithm behind HydroNanoConstruct is described, which has been designed to reproduce the structures of hydrated Metal Oxide surfaces reported in literature^21–26,39,40^ and identified through density functional theory calculations. HydroNanoConstruct’s algorithm processes metal/metalloid atoms based on their coordination number in the bulk phase of the metal/metalloid Oxide. Similarly, it classifies the hydrogen atoms of NPs into three categories: (a) hydrogen atoms bonded to bridging oxygens, (b) hydrogen atoms that are part of hydroxyl groups where the oxygen is bonded to one metal/metalloid atom, and (c) hydrogen atoms that are part of water molecules bonded to a metal/metalloid atom.

## Supplementary Material





## Data Availability

The tool and its manual are freely available via the *EosCloud* Platform at https://eoscloud.entelos.eu/ssbd4chem/nanoconstruct/. The source code is proprietary and not publicly available.

## Notes

The workflow of HydroNanoConstruct is provided as The Supporting Information is available free of charge at https://pubs.acs.org/doi/10.1021/acs.jcim.5c01889.

(A) Workflow, descriptor calculations, and application examples (PDF)
(B) a machine-readable JSON file suitable for computational parsing and automation (PDF)
