# Supplementary material for: HydroNanoConstruct: A Web Application for Digital Construction, Crystal Growth Investigation, and Atomistic Descriptor Calculation of Hydrated Metal Oxide Nanoparticles Powered by the EosCloud Platform
Source: J Chem Inf Model. 2025 Dec 29;66(1):1–6. doi: 10.1021/acs.jcim.5c01889 (PMC12801294; doi:10.1021/acs.jcim.5c01889)
Supplement: Supplementary file 1 [file ci5c01889_si_001.pdf]

Supporting Information for the paper

"HydroNanoConstruct: A Web Application for Digital Construction, Crystal Growth Investigation, and Atomistic Descriptor Calculation of Hydrated Metal Oxide Nanoparticles powered by the EosCloud Platform"

Panagiotis D. Kolokathis[1]*, Anastasios Sourpis[2], Dimitris Mintis[2,3], Andreas Tsoumanis[2,3], Georgia Melagraki[4], Milica Velimirovic[5], Iseult Lynch[2,6] and Antreas Afantitis[2,3,7] *

[1] NovaMechanics MIKE, Piraeus 18545, Greece
[2] Entelos Institute, Larnaca 6059, Cyprus
[3] NovaMechanics Ltd, Nicosia 1070, Cyprus
[4] Division of Physical Sciences and Applications, Hellenic Military Academy, Vari 16672, Greece
[5] Flemish Institute for Technological Research (VITO), Mol, 2400, Belgium
[6] School of Geography, Earth and Environmental Sciences, University of Birmingham, Birmingham B15 2TT, United Kingdom
[7] Department of Pharmacy, Frederick University, Nicosia 1036, Cyprus
*Correspondence: kolokathis@novamechanics.com (P.D Kolokathis), afantitis@novamechanics.com (A. Afantitis)

1. Workflow and algorithm of *HydroNanoConstruct*

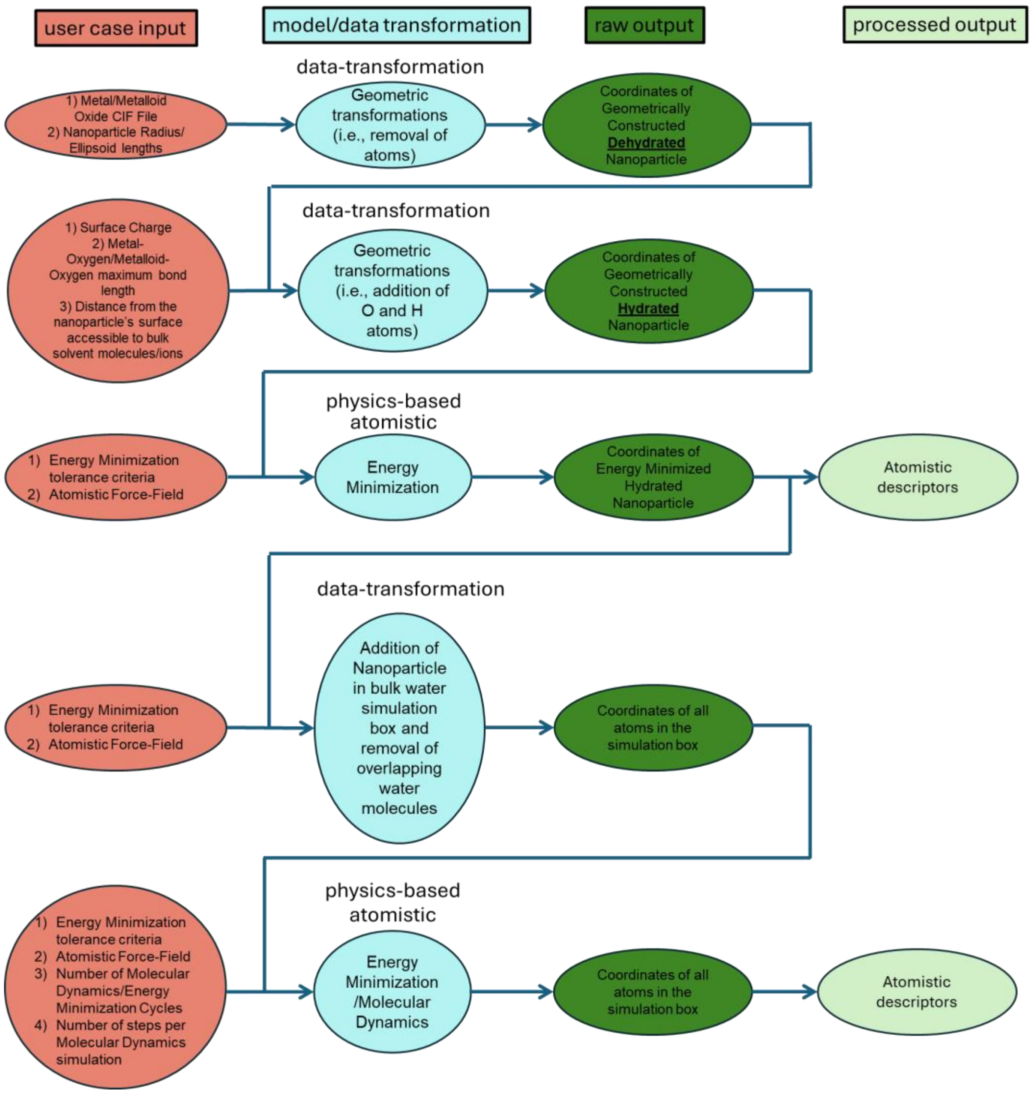

Figure S1. Workflow for the digital construction of hydrated metal/metalloid oxide (MO) nanoparticles (NPs) and its output

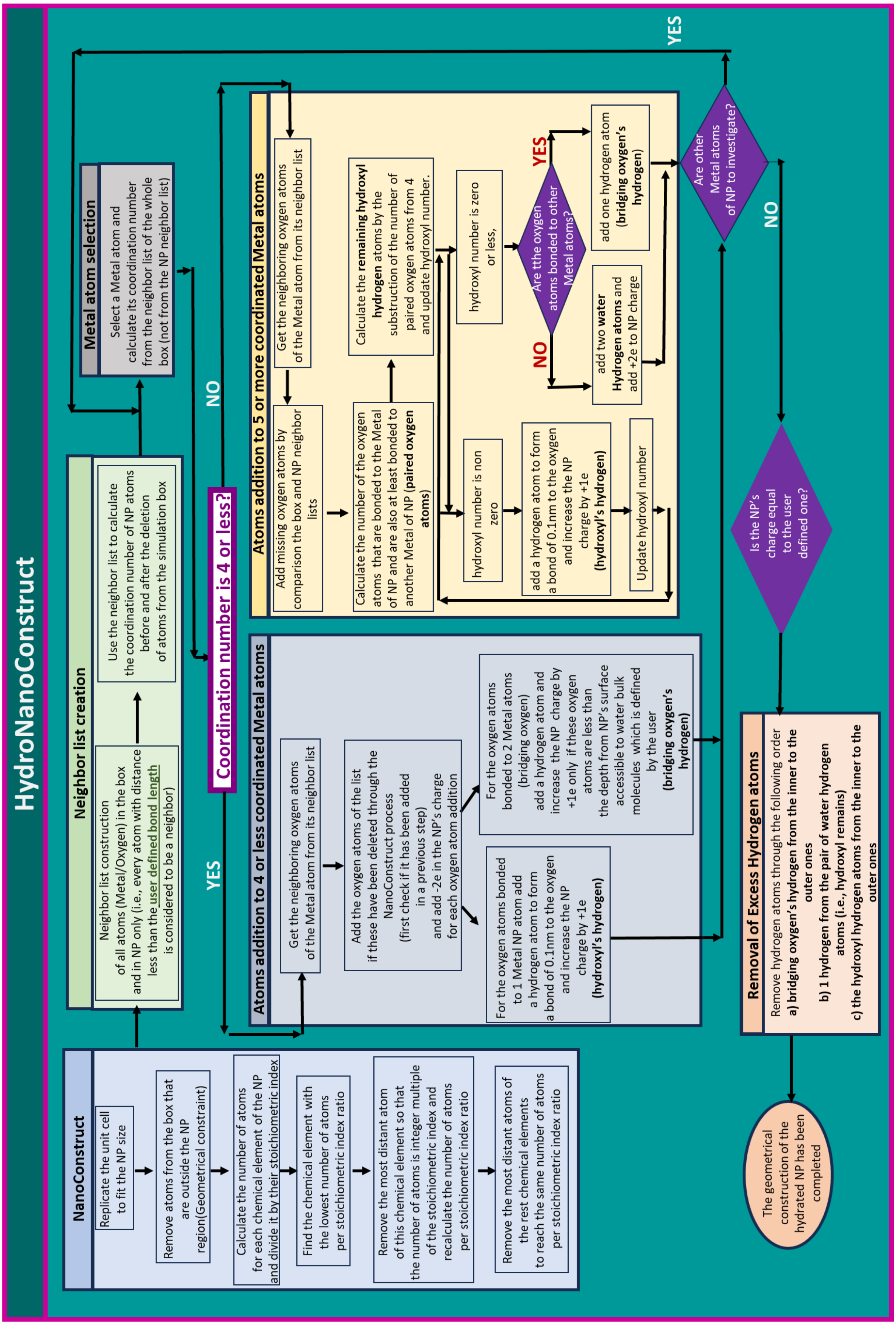

Figure S2: *HydroNanoContruct*'s algorithm for the digital and geometric construction of hydrated MO NPs.

2. Calculation of Atomistic Descriptors and Physicochemical Properties of the NP

In addition to the construction of MO NPs in water, *HydroNanoConstruct* also performs calculations for a set of descriptors that can be used to enrich experimentally obtained descriptors for the development of Machine Learning models. These descriptors are calculated using the atomic coordinates of the dehydrated NP which are also part of the hydrated NP. These descriptors include: a) the maximum ellipsoid length per direction of the NP after the MD-Minimization Cycles which may differ from the initial input values provided by the user, b) the NP's surface area, volume and number of atoms, c) the average potential energy per atom for the bulk water, bulk metal oxide, NP in vacuum and NP in water, d) the static contribution to the surface tension of the NP in water and in vacuum as well as the static contribution to the wetting (=γ_{SG}-(γ_{SL}+γ_{LG})). To calculate the average potential energy per atom for a bulk MO, its unit cell is replicated so that the box is at least 28 Å in each direction. For the calculation of the average potential energy per atom of bulk water, a cubic box with edge length of 37.24 Å is used.

Concerning the calculation of surface tension, Eq. S1 [S1] is used where *F*, *A*, *U*, *T*, *S* and γ represent Helmholtz Free Energy, Surface Area, Internal energy, Temperature, Entropy and Surface Tension respectively (i.e., A_{bulk} is zero because there is no interface for the bulk material by definition). Eq. S1 shows that the surface tension can be divided into a) an energetic γ_U part where the internal energy (or equivalently the average potential energy as the kinetic energy does not change) for a specific temperature of the system is used, and b) an entropic γ_S part where the entropy is needed.

$$\gamma = \left(\frac{\partial F}{\partial A}\right)_{T,V} \cong \left(\frac{F_{\text{NP}} - F_{\text{bulk}}}{A_{\text{NP}} - A_{\text{bulk}}}\right)_{T,V} = \frac{F_{\text{NP}} - F_{\text{bulk}}}{A_{\text{NP}}} = \frac{F_{\text{NP-water}} - F_{\text{bulk,crystal}} - F_{\text{bulk,water}}}{A_{\text{NP}}}$$

$$\xrightarrow{F=U-TS} \gamma \cong \frac{U_{\text{NP-water}} - U_{\text{bulk,crystal}} - U_{\text{bulk,water}}}{A_{\text{NP}}} - T\,\frac{S_{\text{NP-water}} - S_{\text{bulk,crystal}} - S_{\text{bulk,water}}}{A_{\text{NP}}} \qquad \text{(S1)}$$

$$\rightarrow \gamma \cong \gamma_U + \gamma_S$$

Furthermore, the energetic part of the surface tension γ_U can be divided to a) the contribution of the global energy minimum $\gamma_{U,min}$ (static contribution) and b) additional contributions γ_{rest} arising from thermal vibrations especially for crystals and configurational fluctuations for liquids. This separation into two terms is very useful because the entropic part is highly dependent on the temperature.

Due to the high computational cost of the calculation of the internal Energy *U* (especially for reactive Force-Fields or Density Functional theory calculations) through MD simulations, $\gamma_{U,min}$ is calculated instead of γ [S2-S5] and it has been selected to be used as a descriptor in *HydroNanoConstruct*. The energetic surface tension part γ_U of crystalline materials can be approximated using $\gamma_{U,min}$ if deviations from the global energy minimum (i.e., the errors due to thermal fluctuations) are expected to cancel out. This approach is valid for crystals in vacuum (e.g., crystals oscillate around their equilibrium positions) [S2-S7] but it is not valid for crystals surrounded by liquids where configurational fluctuations of the liquid water are not cancelled out [S8, S9].

Based on Eq. 1 and the previous analysis, equilibrated MD simulations are needed for more accurate calculations of the surface tension γ but these simulations are prohibitive for a web

application such as *HydroNanoConstruct* (e.g., due to web server timeouts, limited CPU and memory resources, etc). These MD simulations require special treatment because of a) the absence of dissolved metal ions in the simulation box which could lead to a partial dissolve of the NP until the reaction equilibrium is achieved and b) computational time required to get equilibrated values. Due to these computational barriers, users can download the *HydroNanoConstruct* output files and run them locally to accurately calculate the energetic part of the surface tension γ_U.

Regarding the entropic part of the surface tension γ_U, although the entropy of the bulk metal oxide is assumed unchanged in the NP due to the low mobility of its atoms, the entropic contribution to the surface tension has been found to be significant [S10] primarily due to the chemisorbed water molecules which leads to a decrease in entropy [S10]. This decrease in entropy can be attributed to the reduced freedom of movement of chemisorbed water molecules compared to those in the bulk phase. Hiemstra [S10] showed using the Born equation [S10-S12] that the entropy change after the chemisorption of a water molecule is ΔS_{H2O}=-17J/(mol K) which is slightly smaller than the entropy changes at the freezing point of liquid water to ice (ΔS_{H2O}=-22J/(mol K) [S10]).

The calculation of the NP's surface tension is important because it can shed light on NP's crystal growth processes [S13]. It can also be used to calculate the total Free Energy of a NP-water system as shown in Eq. (2) for a system of constant volume and temperature. In this Equation, Q_r is the reaction quotient, K_{eq} is the equilibrium constant, $N_{reactions}$ is the number of chemical reactions needed to form the NP from the dissolved ions, k_B is the Boltzmann constant, T is the Temperature (i.e., 300K has been selected as a default value for HydroNanoConstruct) and A is the NP's surface area.

$$\Delta F_{\text{tot}} = \Delta F_{\text{bulk}} + \Delta F_{\text{surface}} = N_{\text{reactions}} k_{\text{B}} T \ln\left(\frac{Q_{\text{r}}}{K_{\text{eq}}}\right) + \gamma A \tag{S2}$$

While the surface area A is well-defined for macroscopic structures based on the assumption of continuous matter, its definition becomes unclear at the nanoscale where the concept of continuity no longer applies. The NP's ellipsoid axes are calculated after the equilibration of the NP in water to be used for the calculation of the surface area of the NP [S14]. The ellipsoid axes are obtained by measuring the distance between pairs of atoms belonging to the stoichiometric NP along the *x*, *y* and *z* axis without considering their van der Waals radii.

3. Implementation and Validation of HydroNanoConstruct's Algorithm

A digital construction of NPs during Stage 2 is achieved through the application of the algorithm presented in Figure S1. This algorithm digitally constructs metal and metalloid oxide NPs and aims to generate realistic configurations of them in aqueous environments. It adds oxygen atoms to a stoichiometrically preconstructed NP in order to maintain the coordination number of the metal or metalloid atoms equal to that of their bulk phase. Subsequently, hydrogen atoms are added to the surface oxygen atoms so that the NP gets the user-defined electric charge, which is specified through the graphical user interface (GUI) and depends on the pH of the aqueous solution (see Ref. S19). The resulting NP surface remains structurally consistent with the bulk material while it carries a surface charge compatible with the desired pH.

During the algorithm's procedure, the addition of oxygen atoms initially inserts a high negative charge to the NP due to the incorporation of O^{2-} ions. In the following step, hydrogen atoms are added, producing a temporary high positive charge since the number of hydrogen atoms added is typically greater than twice the number of oxygen atoms (see Figure S2 and Section 3.1 in the main manuscript for more details). If the number of hydrogen atoms is insufficient to reach the user-defined NP charge, a message is displayed to inform the user. Because the algorithm produces structures close to equilibrium, it enables rapid convergence to a stable configuration with significantly fewer MD minimization cycles, assuring the computational stability and efficiency of the web-based application. Even if there were deviations of the structure of the algorithm compared to a structured produced through equilibrated MD simulations with reactive force fields, the algorithm could still remain suitable for generating initial configurations, requiring only additional MD cycles and minimization steps to reach at a realistic structure after the displacement of hydrogen atoms to nearby positions at Stages 3 and 4. To ensure that the constructed NPs are realistic, Stage 3 (Energy minimization with absence of surrounding water molecules) and Stage 4 (MD/Minimization Cycles with presence of surrounding water molecules) were included in addition to Stage 2 (geometrical NP construction). Stage 3 leads to more realistic NPs than Stage 2 because the energy minimization allows the expansion/shrinkage of NP and the displacement of hydrogen and oxygen atoms. Stage 4 is more computationally demanding than Stage 3 but it includes any effect on the NP due to its interaction with the water molecules of the surrounding environment.

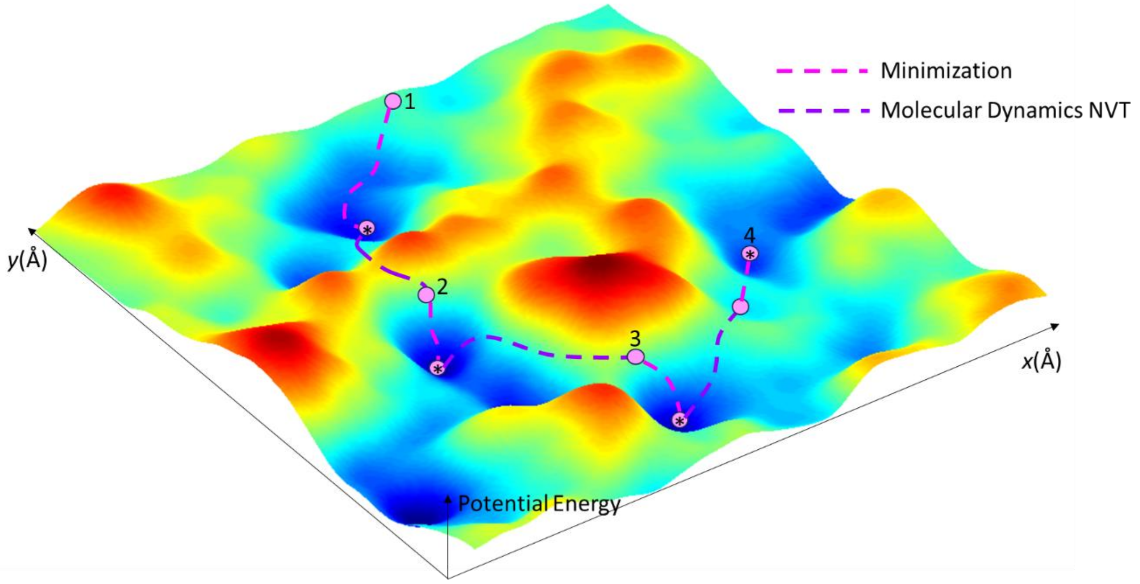

Figure S3. Sketch illustrating the application of MD (purple line)-Minimization (pink line) cycles to sample a rugged Energy Landscape (colormap) in search of the global minimum. Star markers illustrate the local minima of the energy landscape. The sketch has been generated using the summation of 1200 random gaussians (i.e., random width, amplitude and center values in the range from 0 to 1).

The accurate and efficient construction achieved in Stage 2 facilitates rapid equilibration of the system during Stage 3 and Stage 4. Due to the high computational cost of Stage 4 arising from high RAM and CPU demands for systems containing thousands of atoms, even for NPs smaller than 3 nm, *HydroNanoConstruct* currently limits Stage 4 to a maximum of 10 steps per MD run and 5 MD/energy minimization cycles. The accuracy of the algorithm and its ability to generate realistic NP structures were validated using 400 MD-minimization cycles (5 MD steps per cycle, with an energy tolerance criterion of 10^{-4}, defined as the ratio of the energy change between successive iterations to the total energy magnitude). The displacement of hydrogen and oxygen atoms from their initial positions in the geometrically reconstructed NP was found to be less than 0.4% of the added atoms. For instance, only 3 hydrogen atoms were displaced out of a total of 262 oxygen and 524 hydrogen atoms added to a dehydrated Fe_3O_4 (magnetite) NP with a diameter of 3.25 nm and a total of 1,666 atoms. To assess the algorithm's capability to handle various atomic coordination environments, SiO_2 (α-quartz) was used to evaluate its performance in constructing tetrahedrally coordinated systems, while Fe_3O_4 (magnetite) was selected due to the coexistence of tetrahedral and octahedral iron atoms, allowing validation in more complex systems.

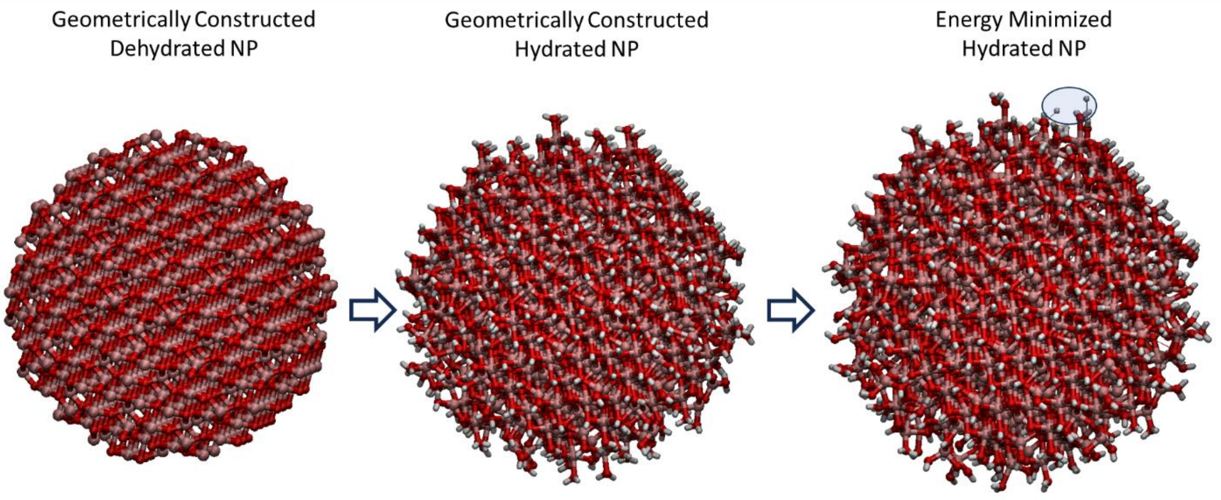

Figure S4: Procedure for construction of Energy Minimised Fe_3O_4 (magnetite) nanoparticle (NP) starting from a dehydrated Fe_3O_4 magnetite NP with diameter equal to 3.25 nm and containing 1666 atoms (left), adding 262 oxygen and 524 hydrogen atoms based on the algorithm of Figure 2 of the main document (middle) and applying 400 MD-Minimization Cycles (i.e., 5 Molecular Dynamics (MD) steps per cycle and energy tolerance criterion for the energy minimization equal to 10^{-4} where the energy tolerance criterion is the energy change between successive iterations divided by the energy magnitude) after the addition of solvent water molecules (right). The circle at the top right of the right Figure shows two hydrogen atoms that have been displaced from their initial positions to create hydronium after their reaction with the solvent water atoms which have been omitted from the Figure for clarity. The CIF file from the COD with entry number 1010369 was used [S15] and the REAXFF [S16] was used. Molecular visualization of Fe_3O_4 (magnetite) NP was performed using VMD (Visual Molecular Dynamics) developed by the Theoretical and Computational Biophysics Group in the Beckman Institute for Advanced Science and Technology at the University of Illinois at Urbana-Champaign [S20].

The potential energy per atom for the bulk, dehydrated and the hydrated NPs of Fe_3O_4 during 400 MD-Minimisation cycles is illustrated in Figures S6-S8 and has been calculated to show that we have explored the potential energy landscape of the simulation system and found its global minimum if we assume that there is not a potential energy barrier that cannot be overcome during the 400 MD-Minimisation cycles. These Figures show that there is a very rugged energy landscape for the Fe_3O_4 NP with diameter 1nm while for the rest sizes the potential energy per atom has small deviations. Sharp drops in potential energy especially at the beginning of MD-Minimization Cycles are illustrated in Figures S6-S8. Figures S6-S8 show that if a user wants to get reliable values of the descriptors, then at least 100 MD-Minimization Cycles are needed to be applied.

In case, a Metal Oxide Nanoparticle is a porous material and water can penetrate inside it, then many (i.e., more than 1000) MD steps may be needed to be selected to be able to incorporate the event. To include the effect of the penetration of the water molecule into the porous of nanoparticles, Stage 2 requires from the user to insert the parameter “Maximum distance from surface to add hydrogen atoms”, which defines the depth of the penetration. This parameter defines the exact region on which the addition of hydrogen atoms will be applied.

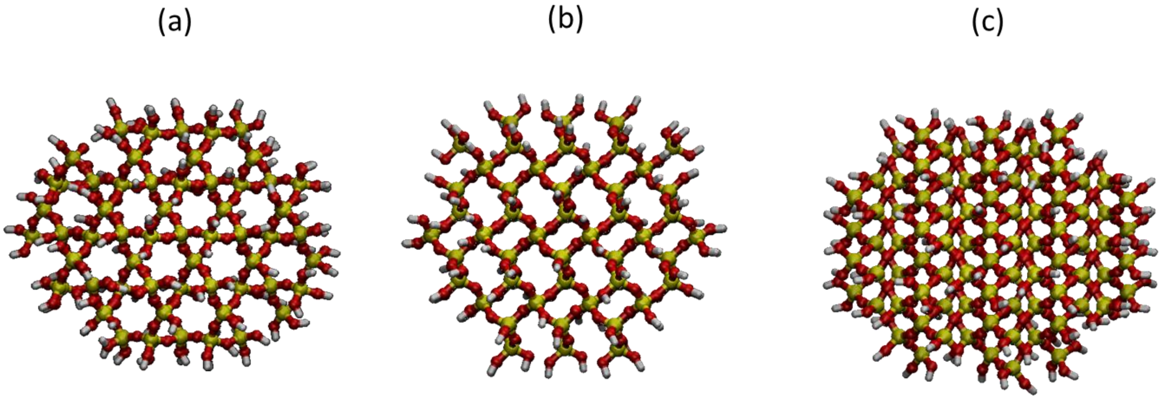

Figure S5. Hydrated SiO_2/a-quartz NPs generated by *HydroNanoConstruct* and their projections at x-y, y-z and x-z planes. The lengths of their ellipsoid axes are (2.5, 2, 2), (2.0, 2.5, 2.0) and (2.0, 2.0, 2.5) nm respectively. The CIF file from COD with entry number 1011159 [S17] and the REAXFF [S18] was used. Molecular visualization of Fe_3O_4 (magnetite) NP was performed using VMD (Visual Molecular Dynamics) developed by the Theoretical and Computational Biophysics Group in the Beckman Institute for Advanced Science and Technology at the University of Illinois at Urbana-Champaign [S20].

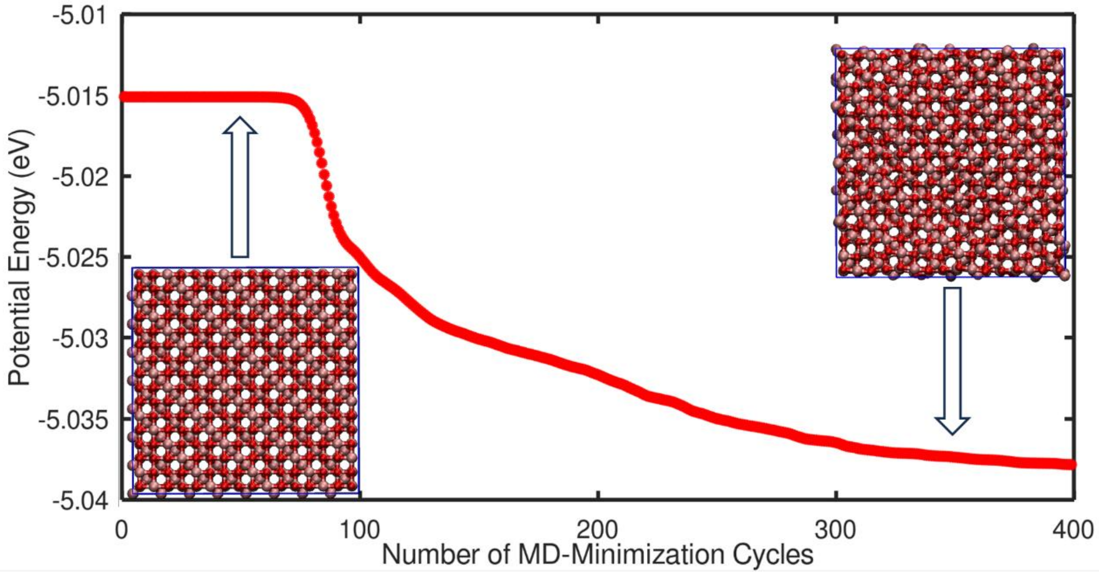

Figure S6. Potential Energy per atom for the bulk phase of Fe_3O_4 using the Force-Field of Aryanpour et al [S18]. Screenshots of the simulation box before (left) and after (right) the 400 MD-Minimization Cycles. The bulk phase of Fe_3O_4 (magnetite) is illustrated at the start of the simulation. Molecular visualization of Fe_3O_4 (magnetite) was performed using VMD (Visual Molecular Dynamics) developed by the Theoretical and Computational Biophysics Group in the Beckman Institute for Advanced Science and Technology at the University of Illinois at Urbana-Champaign [S20].

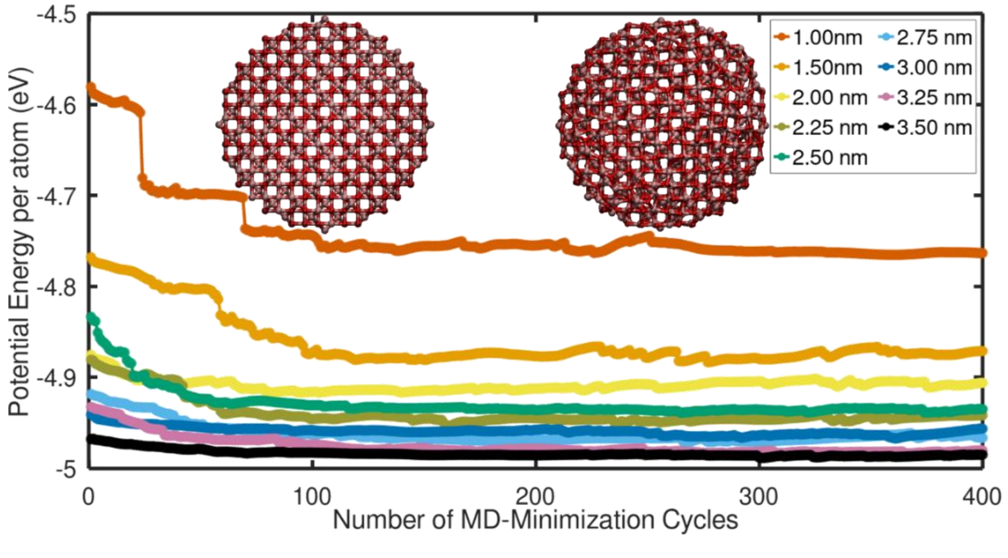

Figure S7. Potential Energy per atom for a range of spherical Fe_3O_4 NPs in vacuum/air. Each color corresponds to the NP diameter mentioned in the legend. A geometrically constructed Fe_3O_4 NP with diameter equal to 3.5 nm (top left) and an energy-minimized one after 400 MD-Minimization Cycles (i.e., 5 MD steps per cycle and energy tolerance criterion for the energy minimization equal to 10^{-4}). Molecular visualization of Fe_3O_4 (magnetite) NP was performed using VMD (Visual Molecular Dynamics) developed by the Theoretical and Computational Biophysics Group in the Beckman Institute for Advanced Science and Technology at the University of Illinois at Urbana-Champaign [S20].

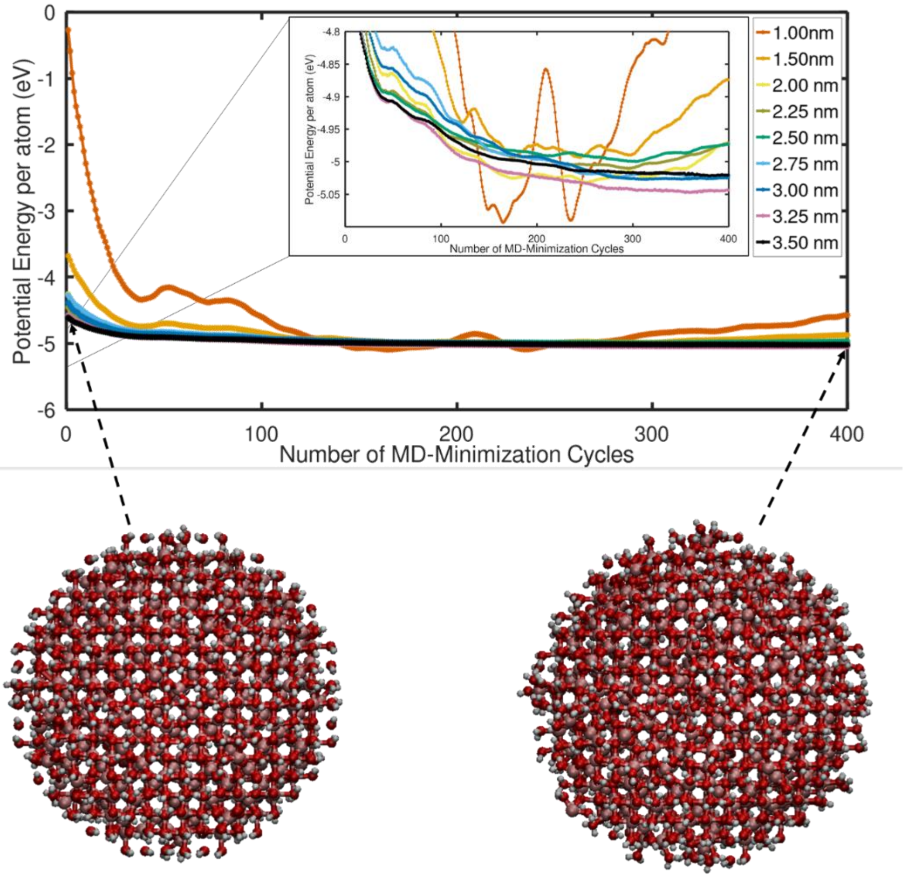

Figure S8. Potential Energy per atom for a range of spherical Fe_3O_4 NPs in water. Each color corresponds to the NP diameter mentioned in the legend. An initially Energy minimised Fe_3O_4 NP with diameter equal to 3.5 nm at the beginning (bottom left) and at the end (bottom right) of 400 MD-Minimization Cycles. Molecular visualization of Fe_3O_4 (magnetite) NP was performed using VMD (Visual Molecular Dynamics) developed by the Theoretical and Computational Biophysics Group in the Beckman Institute for Advanced Science and Technology at the University of Illinois at Urbana-Champaign [S20].

4. References

[S1] Ip, S. W.; Toguri, J. M. The Equivalency of Surface Tension, Surface Energy and Surface Free Energy. *Journal of Materials Science* **1994**, *29* (3), 688–692. https://doi.org/10.1007/BF00445980.

[S2] De Leeuw, N. H.; Cooper, T. G. Surface Simulation Studies of the Hydration of White Rust Fe(OH)2, Goethite α-FeO(OH) and Hematite α-Fe2O3. *Geochimica et Cosmochimica Acta* **2007**, *71* (7), 1655–1673. https://doi.org/10.1016/j.gca.2007.01.002.

[S3] Sassi, M.; Chaka, A. M.; Rosso, K. M. Ab Initio Thermodynamics Reveals the Nanocomposite Structure of Ferrihydrite. *Commun Chem* **2021**, *4* (1), 134. https://doi.org/10.1038/s42004-021-00562-7.

[S4] Stuart, N. M.; Sohlberg, K. A Method of Calculating Surface Energies for Asymmetric Slab Models. *Phys. Chem. Chem. Phys.* **2023**, *25* (19), 13351–13358. https://doi.org/10.1039/D2CP04460A.

[S5] Zhu, Y.; Luo, B.; Sun, C.; Liu, J.; Sun, H.; Li, Y.; Han, Y. Density Functional Theory Study of α-Bromolauric Acid Adsorption on the α-Quartz (1 0 1) Surface. *Minerals Engineering* **2016**, *92*, 72–77. https://doi.org/10.1016/j.mineng.2016.03.007.

[S6] Nyman, J.; Day, G. M. Static and Lattice Vibrational Energy Differences between Polymorphs. *CrystEngComm* **2015**, *17* (28), 5154–5165. https://doi.org/10.1039/C5CE00045A.

[S7] Kundu, T. K.; Rao, K. H.; Parker, S. C. Atomistic Simulation Studies of Magnetite Surface Structures and Adsorption Behavior in the Presence of Molecular and Dissociated Water and Formic Acid. *Journal of Colloid and Interface Science* **2006**, *295* (2), 364–373. https://doi.org/10.1016/j.jcis.2005.09.022.

[S8] Yun, Y.; Khaliullin, R. Z.; Jung, Y. Correlated Local Fluctuations in the Hydrogen Bond Network of Liquid Water. *J. Am. Chem. Soc.* **2022**, *144* (29), 13127–13136. https://doi.org/10.1021/jacs.2c02362.

[S9] Shelton, D. P. Hydrogen Bond Network Modes in Liquid Water. *Phys. Rev. B* **2023**, *108* (17), 174203. https://doi.org/10.1103/PhysRevB.108.174203.

[S10] Hiemstra, T. Formation, Stability, and Solubility of Metal Oxide Nanoparticles: Surface Entropy, Enthalpy, and Free Energy of Ferrihydrite. *Geochimica et Cosmochimica Acta* **2015**, *158*, 179–198. https://doi.org/10.1016/j.gca.2015.02.032.

[S11] Schmid, R.; Miah, A. M.; Sapunov, V. N. A New Table of the Thermodynamic Quantities of Ionic Hydration: Values and Some Applications (Enthalpy–Entropy Compensation and Born Radii). *Phys. Chem. Chem. Phys.* **2000**, *2* (1), 97–102. https://doi.org/10.1039/a907160a.

[S12] Conway, B. E. *Ionic Hydration in Chemistry and Biophysics*; Studies in physical and theoretical chemistry; Elsevier Scientific Pub. Co. : distributors for the U.S. and Canada, Elsevier/North-Holland: Amsterdam ; New York, 1981.

[S13] Polte, J. Fundamental Growth Principles of Colloidal Metal Nanoparticles – a New Perspective. *CrystEngComm* **2015**, *17* (36), 6809–6830. https://doi.org/10.1039/C5CE01014D.

[S14] Kolokathis, P. D.; Zouraris, D.; Voyiatzis, E.; Sidiropoulos, N. K.; Tsoumanis, A.; Melagraki, G.; Tämm, K.; Lynch, I.; Afantitis, A. NanoConstruct: A Web Application Builder of Ellipsoidal Nanoparticles for the Investigation of Their Crystal Growth, Stability, and the Calculation of Atomistic Descriptors. *Computational and Structural Biotechnology Journal* **2024**, *25*, 81–90. https://doi.org/10.1016/j.csbj.2024.05.039.

[S15] Montoro, V., Miscibilita fra gli ossidi salini di ferro e di manganese, *Gazzetta Chimica Italiana* **1938**, 68, 728-733

[S16] Zhang, Y.; Liu, X.; Van Duin, A. C. T.; Lu, X.; Meijer, E. J. Development and Validation of a General-Purpose ReaxFF Reactive Force Field for Earth Material Modeling. *The Journal of Chemical Physics* **2024**, *160* (9), 094103. https://doi.org/10.1063/5.0194486

[S17] Machatschki, F Kristallstruktur von Tiefquarz, *Fortschritte der Mineralogie* **1936**, 20, 45-47

[S18] Aryanpour, M.; Van Duin, A. C. T.; Kubicki, J. D. Development of a Reactive Force Field for Iron−Oxyhydroxide Systems. *J. Phys. Chem. A* **2010**, *114* (21), 6298–6307. https://doi.org/10.1021/jp101332k.

[S19] Parks, G. A. The Isoelectric Points of Solid Oxides, Solid Hydroxides, and Aqueous Hydroxo Complex Systems. *Chem. Rev.* **1965**, *65* (2), 177–198. https://doi.org/10.1021/cr60234a002.

[S20] Humphrey, W., Dalke, A. and Schulten, K., VMD -Visual Molecular Dynamics, *J. Molecular Graphics* **1996**, *14*, pp. 33-38.
