# Supplementary material for: HydroNanoConstruct: A Web Application for Digital Construction, Crystal Growth Investigation, and Atomistic Descriptor Calculation of Hydrated Metal Oxide Nanoparticles Powered by the EosCloud Platform
Source: J Chem Inf Model. 2025 Dec 29;66(1):1–6. doi: 10.1021/acs.jcim.5c01889 (PMC12801294; doi:10.1021/acs.jcim.5c01889)
Supplement: Supplementary file 2 [file ci5c01889_si_002.pdf]

```
{
  "inputs": {
    "Structure File": "Metal Oxide CIF File",
    "Nanoparticle Geometry": {
      "Radius": "value",
      "Ellipsoid Lengths": "values"
    },
    "Surface Properties": {
      "Charge": "value",
      "Max Metal-Oxygen Bond Length": "value",
      "Solvent Accessibility": "value"
    },
    "Simulation Parameters": {
      "Energy Minimization Tolerance": "value",
      "Force Field": "Atomistic Force Field",
      "MD Cycles": "value",
      "MD Timestep": "value"
    }
  },
  "steps": [
    {
      "name": "Geometric Transformation - Dehydration",
      "function": "geometric_transform_dehydration",
      "inputs": [
        "Structure File",
        "Nanoparticle Geometry"
      ],
      "output": "Dehydrated Nanoparticle"
    },
    {
      "name": "Geometric Transformation - Hydration",
      "function": "geometric_transform_hydration",
      "inputs": [
        "Dehydrated Nanoparticle",
        "Surface Properties"
      ],
      "output": "Hydrated Nanoparticle"
    },
    {
      "name": "Energy Minimization",
      "function": "energy_minimization",
      "inputs": [
        "Hydrated Nanoparticle",
        "Force Field",
        "Energy Minimization Tolerance"
      ],
      "output": "Energy Minimized Hydrated Nanoparticle"
    },
    {
      "name": "Add Nanoparticle to Water Box",
      "function": "add_nanoparticle_to_water_box",
```

```
      "inputs": [
        "Energy Minimized Hydrated Nanoparticle"
      ],
      "options": {
        "remove_overlaps": true
      },
      "output": "Solvated System"
    },
    {
      "name": "Run MD Simulation",
      "function": "run_md_simulation",
      "inputs": [
        "Solvated System",
        "Force Field",
        "Energy Minimization Tolerance",
        "MD Cycles",
        "MD Timestep"
      ],
      "output": "Final Simulated Structure"
    }
  ],
  "outputs": {
    "raw_outputs": {
      "Dehydrated Nanoparticle": "coordinates",
      "Hydrated Nanoparticle": "coordinates",
      "Energy Minimized Hydrated Nanoparticle": "coordinates",
      "Solvated System": "coordinates",
      "Final Simulated Structure": "coordinates"
    },
    "processed_outputs": {
      "Descriptors_Minimized": "computed from Energy Minimized Hydrated
Nanoparticle",
      "Descriptors_MD": "computed from Final Simulated Structure"
    }
  }
}
```
